# Impact of a commercially available model-based dose calculation algorithm on treatment planning of high-dose-rate brachytherapy in patients with cervical cancer

**DOI:** 10.1093/jrr/rrx081

**Published:** 2018-01-25

**Authors:** Kota Abe, Noriyuki Kadoya, Shinya Sato, Shimpei Hashimoto, Yujiro Nakajima, Yuya Miyasaka, Kengo Ito, Rei Umezawa, Takaya Yamamoto, Noriyoshi Takahashi, Ken Takeda, Keiichi Jingu

**Affiliations:** 1 Department of Radiation Oncology, Tohoku University Graduate School of Medicine, 1-1 Seiryo-machi, Aoba-ku, Sendai, 980-8574, Japan; 2 Department of Radiation Oncology, Tokyo Metropolitan Cancer and Infectious Diseases Center Komagome Hospital, 3-18-22, Honkomagome, Bunkyo-ku, Tokyo, 113-8677, Japan; 3 Department of Radiation Oncology, Yamagata University Faculty of Medicine, 2-2-2, Iidani, Yamagata, 990-9585, Japan; 4 Department of Therapeutic Radiology, Tohoku University Graduate School of Medicine, 1-1 Seiryo-machi, Aoba-ku, Sendai, 980-8574, Japan

**Keywords:** radiotherapy, brachytherapy, model-based dose calculation algorithm, Monte Carlo simulation, cervical cancer

## Abstract

We evaluated the impact of model-based dose calculation algorithms (MBDCAs) on high-dose-rate brachytherapy (HDR-BT) treatment planning for patients with cervical cancer. Seven patients with cervical cancer treated using HDR-BT were studied. Tandem and ovoid applicators were used in four patients, a vaginal cylinder in one, and interstitial needles in the remaining two patients. MBDCAs were applied to the Advanced Collapsed cone Engine (ACE; Elekta, Stockholm, Sweden). All plans, which were originally calculated using TG-43, were re-calculated using both ACE and Monte Carlo (MC) simulations. Air was used as the rectal material. The mean difference in the rectum D_2cm^3^_ between ACE_rec-air_ and MC_rec-air_ was 8.60 ± 4.64%, whereas that in the bladder D_2cm^3^_ was −2.80 ± 1.21%. Conversely, in the small group analysis (*n* = 4) using water instead of air as the rectal material, the mean difference in the rectum D_2cm^3^_ between TG-43 and ACE_rec-air_ was 11.87 ± 2.65%, whereas that between TG-43 and ACE_rec-water_ was 0.81 ± 2.04%, indicating that the use of water as the rectal material reduced the difference in D_2cm^3^_ between TG-43 and ACE. Our results suggested that the differences in the dose–volume histogram (DVH) parameters of TG-43 and ACE were large for the rectum when considerable air (gas) volume was present in it, and that this difference was reduced when the air (gas) volume was reduced. Also, ACE exhibited better dose calculation accuracy than that of TG-43 in this situation. Thus, ACE may be able to calculate the dose more accurately than TG-43 for HDR-BT in treating cervical cancers, particularly for patients with considerable air (gas) volume in the rectum.

## INTRODUCTION

Brachytherapy (BT) has played an essential role in the treatment of gynecological malignancies for decades. Locally advanced cervical cancers have been treated with a combination of concomitant chemotherapy, external beam radiotherapy, and BT boost to the cervical regions [1–5]. Over recent years,3D image-guided BT (3D-IGBT) has been widely employed for treating cervical cancers, resulting in dose–volume histogram (DVH)-based evaluation [1, 3, 6, 7]. Recommendations for the use of 3D-IGBT in patients with cervical cancer were published by the working group for gynecologic brachytherapy of the Groupe Européen de Curiethérapie-European Society for Radiotherapy and Oncology (GEC-ESTRO) and have become a standard practice in many institutions [8–11].

Due to the expansion of 3D-IGBT, there is growing concern regarding the dose calculation accuracy. The recommendations of the American Association of Physicists in Medicine (AAPM) Task Group 43 (TG-43) are commonly used for dose calculation of BT in clinical practice [12, 13]. The dosimetry parameters used in TG-43 are obtained for a single BT source located at the center of a fixed-volume, homogeneous, liquid-water phantom. As a result, this method cannot consider the effect of patients’ body shape and the presence of materials other than water; however, a growing number of papers have demonstrated the non-negligible effects of these variables on BT dose calculation.

To tackle this issue, a model-based dose calculation algorithm (MBDCA) has been gradually introduced as an alternative dose calculation method for BT [14]. AAPM has published Task Group 186, which guides beginners regarding MBDCAs for BT dose calculations and ensures uniformity in practice. Previous papers have already demonstrated the efficacy of MBDCAs for phantoms and for several treatment sites, such as breast tissue [15–19]. However, to date, there has been minimal evaluation of the impact of MBDCAs on the treatment of cervical cancer, particularly for the Advanced Collapsed cone Engine (ACE) method [16, 20].

Therefore, in the present study, we evaluated the impact of ACE on high-dose-rate (HDR)-BT in patients with cervical cancer, in comparison with TG-43 and Monte Carlo (MC) simulation methods.

## MATERIALS AND METHODS

### Patient characteristics

This study received approval from our institutional reviewer board (2017-1-419). Seven patients with cervical cancer who were treated with HDR-BT were included in this study. All the patients received ^192^Ir HDR-BT each week for four consecutive weeks. Tandem and ovoid applicators were used in four patients (with rectum retractors in two), a vaginal cylinder in one patient, and interstitial needles in the remaining two patients. The minimum doses delivered to 90% of the most irradiated volume of the high-risk clinical target volume (CTV) (D90 HR-CTV) and to $D_{2{\mathrm{cm}}^{3}}$ of the rectum and bladder were calculated. At least 6 Gy dose was prescribed for the D90 HR-CTV in each BT session. The dose constraint was 75 Gy in $D_{2{\mathrm{cm}}^{3}}$ for the rectum and 90 Gy in $D_{2{\mathrm{cm}}^{3}}$ for the bladder. The treatment planning system used for BT was Oncentra version 4.1 (Elekta, Stockholm, Sweden), and this was used to design the CT-based treatment plan. The dose calculation in normal clinical practice is performed using the TG-43 method.

### Calculations for MBDCA and MC simulations

All plans, originally calculated using the TG-43 method, were re-calculated using MBDCA and MC simulation methods. In this study, ACE implemented in the Oncentra system was used as the MBDCA. ACE calculates the dose as the sum of the contributions from primary photons, once-scattered photons, and any residual scattering. The primary dose was calculated using a ray trace of the primary photons in a grid that generates scatter energy, which is then input into the collapsed cone superposition convolution algorithm. This algorithm uses angular discretization of radiation transport directions and pre-calculated dose deposition point kernels in water, scaled to reflect the influence of inhomogeneities [21–23]. A material composition and density were assigned to each structure in order to use the MBDCA. The material definition was based on AAPM TG-186 [14]. The patient’s soft tissue was modeled as homogeneous liquid water because this assumption is known to be reasonably accurate for the ^192^Ir photon energy spectrum [16, 24, 25]. In addition, the tandem and ovoid applicator and rectal retractor were modeled as polyphenylsulfone. The material composition and density of air was assigned to the structure of the rectum, based on the assumption that a considerable air (gas) volume exists in the rectum. For comparison, a different assignment was used for the rectum in four patients (Cases 1, 3, 4, and 5): the material composition and density of water was assigned in these cases, based on the assumption that a considerable amount of feces may exist in the rectum. These ACE settings were defined as ACE_rec-air_ and ACE_rec-water_, respectively.

EGSnrc was used as the MC simulation package [26, 27]. This code has been previously used for dosimetric studies of BT [26, 27]. The source geometry was modeled on microselectron HDR v2 [26]. Photon and electron cut-off energies were 10 keV and 512 keV, respectively. The statistical error of MC simulation (average standard deviation of the calculated dose) was <2% (close to simulated sources). Two different settings were used for the material definition of the rectum in the MC simulation method, similar to those used in ACE: air (MC_rec-air_) and water (MC_rec-water_). To commission the EGS code used in this study, it was confirmed that our data were in good agreement with those published by Taylor *et al.* in terms of anisotropy function values in the 80-cm^3^ phantom (<0.5%) [26].

We compared the following clinical DVH parameters calculated by the TG-43, ACE and MC simulations: D90 for HR-CTV; and $D_{2{\mathrm{cm}}^{3}}$, $D_{1{\mathrm{cm}}^{3}}$ and $D_{0.1{\mathrm{cm}}^{3}}$ of the rectum and bladder. The overall percentage differences of DVH parameters between TG-43, ACE and MC simulations were calculated with respect to the MC simulation as the golden standard based on the previous paper about MC dose calculation accuracy [28, 29].

### Statistical analysis

We used the paired *t*-test to determine the significant error (*P* < 0.05) in differences for each parameter. Statistics were generated using JMP Pro 13.0 (SAS Institute Inc., Cary, NC).

## RESULTS

**Fig. 1.**
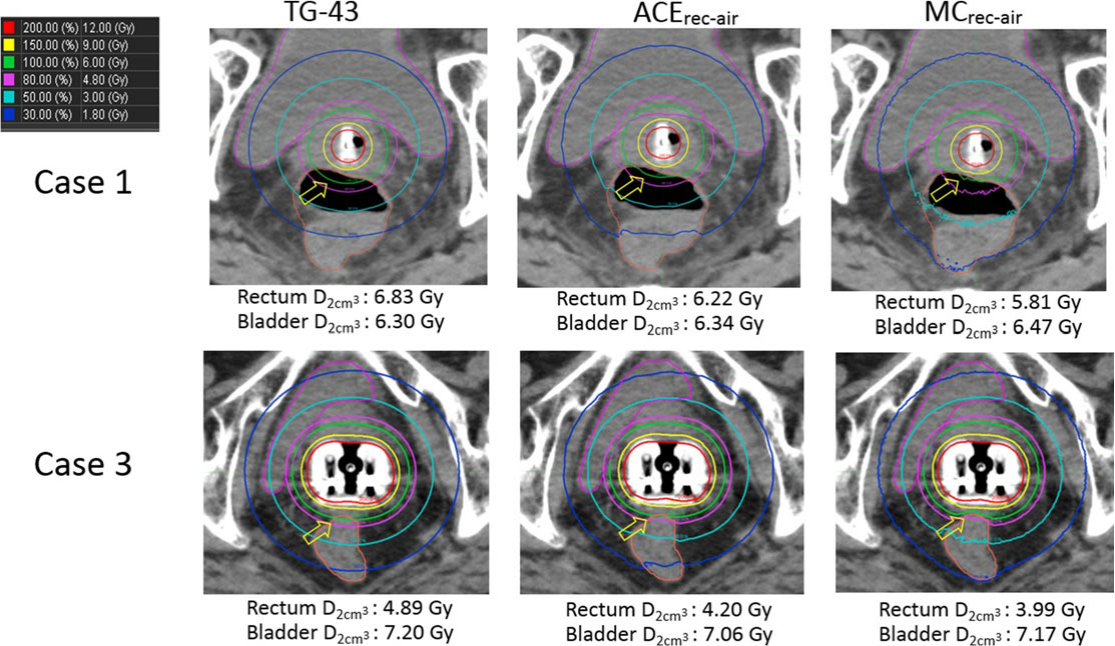
Three dose distributions calculated using TG-43, ACE, and MC methods for Case 1 (tandem and ovoid applicator) and Case 3 (tandem and ovoid applicator with rectal retractor). The 100% and 80% isodose lines of TG-43 and ACE_rec-air_ differ from that of MC_rec-air_ (indicated by yellow arrows).

**Fig. 2.**
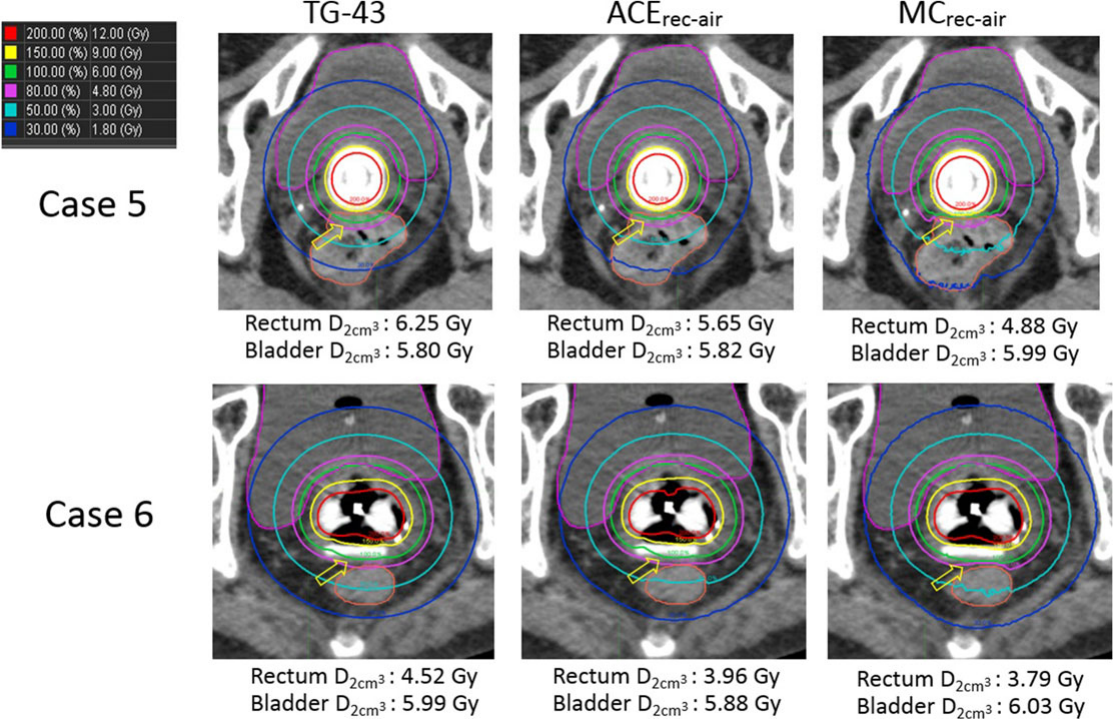
Three dose distributions calculated using TG-43, ACE and MC methods for Case 5 (vaginal cylinder) and Case 6 (tandem and ovoid applicator with rectal retractor + 2 interstitial needles). The 100% and 80% isodose lines of TG-43 and ACE_rec-air_ differ from that of MC_rec-air_ (indicated by yellow arrows).

Figure 1 shows three dose distributions calculated using TG-43, ACE_rec-air_ and MC_rec-air_ for Cases 1 and 3. On visual inspection, the 100% and 80% isodose lines of TG-43 and ACE_rec-air_ differ from those of MC_rec-air_ (indicated by yellow arrows in Fig. 1). Although a moderate difference was observed in the rectum $D_{2{\mathrm{cm}}^{3}}$ between ACE_rec-air_ and MC_rec-air_, ACE_rec-air_ was in better agreement with MC_rec-air_ than with TG-43 (e.g. Case 3: 5.26% for ACE_rec-air_ vs 22.56% for TG-43). For the bladder, both TG-43 and ACE_rec-air_ were in good agreement with MC_rec-air_ (difference <5%). Figure 2 shows the results for Cases 5 and 6. Although a vaginal cylinder was used in Case 5 and an interstitial needle in Case 6, their results were similar to those acquired with a tandem and ovoid applicator, shown in Fig. 1. Table 1 shows the summary of DVH parameters of the rectum and bladder for individual cases. From these results, it can be inferred that the differences in the DVH parameters of TG-43 and ACE_rec-air_ were large for the rectum but small for the bladder. In addition, ACE_rec-air_ showed better agreement with MC_rec-air_ than with TG-43 in terms of the DVH parameters of the rectum. However, some patients showed relatively large differences between these parameters (e.g. Case 5: 15.78% for rectum $D_{2{\mathrm{cm}}^{3}}$). The overall results are shown in Table 2. The mean difference in the rectum $D_{2{\mathrm{cm}}^{3}}$ between TG-43 and ACE_rec-air_ was 11.92 ± 2.25%, whereas that in the D90 for HR-CTV and the bladder $D_{2{\mathrm{cm}}^{3}}$ was 0.81 ± 1.37% and 0.51 ± 1.11%, respectively, indicating a larger difference for the rectum than for the bladder and HR-CTV. In addition, the bladder and D90 for HR-CTV, ACE_rec-air_ was in good agreement with MC_rec-air_ (difference <5%), whereas the mean difference in the rectum $D_{2{\mathrm{cm}}^{3}}$ between ACE_rec-air_ and MC_rec-air_ was 8.60 ± 4.64%, indicating a larger difference for the rectum than for the bladder and HR-CTV. In addition, the mean difference in the rectum $D_{2{\mathrm{cm}}^{3}}$ between TG-43 and MC_rec-air_ was 21.49 ± 4.47%, whereas that between ACE_rec-air_ and MC_rec-air_ was 8.60 ± 4.64%, thus suggesting that ACE_rec-air_ provided a more accurate dose distribution than that obtained by TG-43 when air was used to define the rectal material.

**Table 1.** Summary of DVH parameters for rectum and bladder calculated by the TG-43 and ACE and Monte Carlo simulations for each case

Case	Irradiation technique	DVH parameter		TG43	ACE_rec-air_	MC_rec-air_	TG43-ACE_rec-air_	TG43-MC_rec-air_	ACE_rec-air_-MC_rec-air_
							Diff %	Diff %	Diff %
Case 1	tandem and ovoid applicator	rectum	$D_{0.1{\mathrm{cm}}^{3}}$	9.32	8.64	7.72	7.87	20.73	11.92
			$D_{1.0{\mathrm{cm}}^{3}}$	7.44	6.82	6.26	9.09	18.85	8.95
			$D_{2.0{\mathrm{cm}}^{3}}$	6.83	6.22	5.81	9.81	17.56	7.06
		bladder	$D_{0.1{\mathrm{cm}}^{3}}$	8.87	8.99	9.20	−1.33	−3.59	−2.28
			$D_{1.0{\mathrm{cm}}^{3}}$	6.93	7.03	7.14	−1.42	−2.94	−1.54
			$D_{2.0{\mathrm{cm}}^{3}}$	6.30	6.34	6.47	−0.63	−2.63	−2.01
Case 2	tandem and ovoid applicator	rectum	$D_{0.1{\mathrm{cm}}^{3}}$	7.32	6.77	5.55	8.12	31.89	21.98
			$D_{1.0{\mathrm{cm}}^{3}}$	6.24	5.66	4.84	10.25	28.93	16.94
			$D_{2.0{\mathrm{cm}}^{3}}$	5.76	5.21	4.51	10.56	27.72	15.52
		bladder	$D_{0.1{\mathrm{cm}}^{3}}$	9.16	9.31	9.51	−1.61	−3.68	−2.10
			$D_{1.0{\mathrm{cm}}^{3}}$	7.95	8.03	8.24	−1.00	−3.52	−2.55
			$D_{2.0{\mathrm{cm}}^{3}}$	7.35	7.42	7.60	−0.94	−3.29	−2.37
Case 3	tandem and ovoid applicator with rectal retractor	rectum	$D_{0.1{\mathrm{cm}}^{3}}$	6.58	5.80	5.08	13.45	29.53	14.17
			$D_{1.0{\mathrm{cm}}^{3}}$	5.47	4.70	4.35	16.38	25.75	8.05
			$D_{2.0{\mathrm{cm}}^{3}}$	4.89	4.20	3.99	16.43	22.56	5.26
		bladder	$D_{0.1{\mathrm{cm}}^{3}}$	8.79	8.62	8.83	1.97	−0.45	−2.38
			$D_{1.0{\mathrm{cm}}^{3}}$	7.59	7.46	7.58	1.74	0.13	−1.58
			$D_{2.0{\mathrm{cm}}^{3}}$	7.20	7.06	7.17	1.98	0.42	−1.53
Case 4	tandem and ovoid applicator with rectal retractor	rectum	$D_{0.1{\mathrm{cm}}^{3}}$	7.02	6.63	5.80	5.88	21.03	14.31
			$D_{1.0{\mathrm{cm}}^{3}}$	5.95	5.42	4.93	9.78	20.69	9.94
			$D_{2.0{\mathrm{cm}}^{3}}$	5.52	4.99	4.62	10.62	19.48	8.01
		bladder	$D_{0.1{\mathrm{cm}}^{3}}$	8.38	8.41	8.59	−0.36	−2.44	−2.10
			$D_{1.0{\mathrm{cm}}^{3}}$	7.37	7.36	7.55	0.14	−2.38	−2.52
			$D_{2.0{\mathrm{cm}}^{3}}$	7.06	7.03	7.23	0.43	−2.35	−2.77
Case 5	vaginal cylinder	rectum	$D_{0.1{\mathrm{cm}}^{3}}$	7.70	7.34	6.22	4.90	23.79	18.01
			$D_{1.0{\mathrm{cm}}^{3}}$	6.66	6.08	5.16	9.54	29.07	17.83
			$D_{2.0{\mathrm{cm}}^{3}}$	6.25	5.65	4.88	10.62	28.07	15.78
		bladder	$D_{0.1{\mathrm{cm}}^{3}}$	6.93	7.03	7.22	−1.42	−4.02	−2.63
			$D_{1.0{\mathrm{cm}}^{3}}$	6.12	6.16	6.35	−0.65	−3.62	−2.99
			$D_{2.0{\mathrm{cm}}^{3}}$	5.80	5.82	5.99	−0.34	−3.17	−2.84
Case 6	tandem and ovoid applicator with rectal retractor + 2 interstitial needles	rectum	$D_{0.1{\mathrm{cm}}^{3}}$	6.08	5.40	4.74	12.59	28.27	13.92
			$D_{1.0{\mathrm{cm}}^{3}}$	4.95	4.35	4.05	13.79	22.22	7.41
			$D_{2.0{\mathrm{cm}}^{3}}$	4.52	3.96	3.79	14.14	19.26	4.49
		bladder	$D_{0.1{\mathrm{cm}}^{3}}$	6.87	6.77	6.91	1.48	−0.58	−2.03
			$D_{1.0{\mathrm{cm}}^{3}}$	6.29	6.19	6.34	1.62	−0.79	−2.37
			$D_{2.0{\mathrm{cm}}^{3}}$	5.99	5.88	6.03	1.87	−0.66	−2.49
Case 7	10 interstitial needles	rectum	$D_{0.1{\mathrm{cm}}^{3}}$	7.01	6.36	5.64	10.22	24.29	12.77
			$D_{1.0{\mathrm{cm}}^{3}}$	5.99	5.40	5.09	10.93	17.68	6.09
			$D_{2.0{\mathrm{cm}}^{3}}$	5.64	5.07	4.87	11.24	15.81	4.11
		Bladder	$D_{0.1{\mathrm{cm}}^{3}}$	10.67	10.43	11.12	2.30	−4.05	−6.21
			$D_{1.0{\mathrm{cm}}^{3}}$	7.34	7.25	7.71	1.24	−4.80	−5.97
			$D_{2.0{\mathrm{cm}}^{3}}$	6.68	6.60	6.99	1.21	−4.43	−5.58

**Table 2.** Summary of DVH parameters for the rectum and bladder calculated by the TG-43 and ACE and Monte Carlo simulations in all cases

DVH parameter		TG43	ACE_rec-air_	MC_rec-air_	TG43-ACE_rec-air_			TG43-MC_rec-air_			ACE_rec-air_-MC_rec-air_		
		mean ± SD (Gy)			Diff % ± SD	Range (%)	*P* value	Diff % ± SD	Range (%)	*P* value	Diff % ± SD	Range (%)	*P* value
Rectum	$D_{0.1{\mathrm{cm}}^{3}}$	7.29 ± 0.96	6.71 ± 0.99	5.82 ± 0.89	9.01 ± 2.99	4.9 to 13.45	*P* < 0.001	25.65 ± 3.99	20.73 to 31.89	*P* < 0.001	15.3 ± 3.25	11.92 to 21.98	*P* < 0.001
	$D_{1.0{\mathrm{cm}}^{3}}$	6.1 ± 0.74	5.49 ± 0.76	4.95 ± 0.65	11.39 ± 2.49	9.09 to 16.38	*P* < 0.001	23.31 ± 4.31	17.68 to 29.07	*P* < 0.001	10.74 ± 4.35	6.09 to 17.83	0.001
	$D_{2.0{\mathrm{cm}}^{3}}$	5.63 ± 0.72	5.04 ± 0.72	4.64 ± 0.61	11.92 ± 2.25	9.81 to 16.43	*P* < 0.001	21.49 ± 4.47	15.81 to 28.07	*P* < 0.001	8.6 ± 4.64	4.11 to 15.78	0.004
Bladder	$D_{0.1{\mathrm{cm}}^{3}}$	8.52 ± 1.22	8.51 ± 1.18	8.77 ± 1.32	0.15 ± 1.59	−1.61 to 2.3	0.796	−2.69 ± 1.46	−4.05 to −0.45	0.006	−2.82 ± 1.4	−6.21 to −2.03	0.009
	$D_{1.0{\mathrm{cm}}^{3}}$	7.08 ± 0.63	7.07 ± 0.63	7.27 ± 0.66	0.24 ± 1.21	−1.42 to 1.74	0.670	−2.56 ± 1.59	−4.8 to 0.13	0.009	−2.79 ± 1.39	−5.97 to −1.54	0.003
	$D_{2.0{\mathrm{cm}}^{3}}$	6.63 ± 0.57	6.59 ± 0.57	6.78 ± 0.58	0.51 ± 1.11	−0.94 to 1.98	0.319	−2.3 ± 1.53	−4.43 to 0.42	0.010	−2.8 ± 1.21	−5.58 to −1.53	0.002

**Fig. 3.**
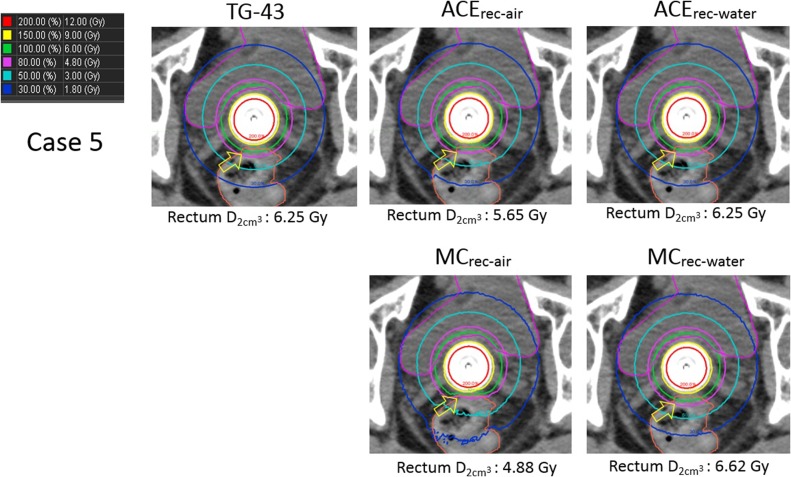
Five dose distributions for Case 5 (vaginal cylinder) were calculated using the following methods: TG-43, ACE with air as the rectal material, ACE with water as the rectal material, MC with air as the rectal material, and MC with water as the rectal material. The 100% and 80% isodose lines of TG-43 and ACE differ from that of MC (indicated by yellow arrows).

Next, Fig. 3 shows the five dose distributions calculated using TG-43, ACE_rec-air_, ACE_rec-water_, MC_rec-air_ and MC_rec-water_ for Case 5. The dose distribution with TG-43 was considerably similar to that with ACE_rec-water_. On the other hand, the 100% and 80% isodose lines of ACE_rec-air_ differ from those of ACE_rec-water_. In addition, the difference in dose distribution between ACE_rec-water_ and MC_rec-water_ was smaller than that between ACE_rec-air_ and MC_rec-air_. A summary of the DVH parameters for the rectum using a different material definition in individual cases is shown in Table 3 and for all cases in Table 4. The mean difference in the rectum $D_{2{\mathrm{cm}}^{3}}$ between TG-43 and ACE_rec-air_ was 11.87 ± 2.65%, whereas that between TG-43 and ACE_rec-water_ was 0.81 ± 2.04%, showing that using water as the rectal material reduced the difference between TG-43 and ACE.

**Table 3.** Summary of DVH parameters for the rectum and bladder calculated by the TG-43 and ACE and Monte Carlo simulations using different definitions of material for the rectum in each case

Patient	Irradiation technique	DVH parameter		TG43	ACE_rec-air_	ACE_rec-water_	MC_rec-air_	MC_rec-water_	TG43-ACE_rec-air_	TG43-ACE_rec-water_	TG43-MC_rec-air_	ACE_rec-air_-MC_rec-air_	ACE_rec-water_-MC_rec-water_
									Diff %	Diff %	Diff %	Diff %	Diff %
Case 1	tandem and ovoid applicator	rectum	$D_{0.1{\mathrm{cm}}^{3}}$	9.32	8.64	9.48	7.72	10.29	7.87	−1.69	20.73	11.92	−7.87
			$D_{1.0{\mathrm{cm}}^{3}}$	7.44	6.82	7.53	6.26	8.18	9.09	−1.20	18.85	8.95	−7.95
			$D_{2.0{\mathrm{cm}}^{3}}$	6.83	6.22	6.90	5.81	7.46	9.81	−1.01	17.56	7.06	−7.51
Case 3	tandem and ovoid applicator with rectal retractor	rectum	$D_{0.1{\mathrm{cm}}^{3}}$	6.58	5.80	6.33	5.08	6.66	13.45	3.95	29.53	14.17	−4.95
			$D_{1.0{\mathrm{cm}}^{3}}$	5.47	4.70	5.24	4.35	5.50	16.38	4.39	25.75	8.05	−4.73
			$D_{2.0{\mathrm{cm}}^{3}}$	4.89	4.20	4.69	3.99	4.92	16.43	4.26	22.56	5.26	−4.67
Case 4	tandem and ovoid applicator with rectal retractor	rectum	$D_{0.1{\mathrm{cm}}^{3}}$	7.02	6.63	7.05	5.80	7.29	5.88	−0.43	21.03	14.31	−3.29
			$D_{1.0{\mathrm{cm}}^{3}}$	5.95	5.42	5.96	4.93	6.19	9.78	−0.17	20.69	9.94	−3.72
			$D_{2.0{\mathrm{cm}}^{3}}$	5.52	4.99	5.52	4.62	5.73	10.62	0.00	19.48	8.01	−3.66
Case 5	vaginal cylinder	rectum	$D_{0.1{\mathrm{cm}}^{3}}$	7.70	7.34	7.78	6.22	8.96	4.90	−1.03	23.79	18.01	−13.17
			$D_{1.0{\mathrm{cm}}^{3}}$	6.66	6.08	6.67	5.16	7.11	9.54	−0.15	29.07	17.83	−6.19
			$D_{2.0{\mathrm{cm}}^{3}}$	6.25	5.65	6.25	4.88	6.62	10.62	0.00	28.07	15.78	−5.59

**Table 4.** Summary of DVH parameters for the rectum and bladder calculated by the TG-43 and ACE and Monte Carlo simulations using different definitions of material for the rectum for all cases

DVH parameter		TG43	ACE_rec-air_	ACE_rec-water_	MC_rec-air_	MC_rec-water_	TG43-ACE_rec-air_		TG43-ACE_rec-water_		TG43-MC_rec-air_		ACE_rec-air_-MC_rec-air_		ACE_rec-water_-MC_rec-water_	
		mean ± SD (Gy)					Diff % ± SD	Range (%)	Diff % ± SD	Range (%)	Diff % ± SD	Range (%)	Diff % ± SD	Range (%)	Diff % ± SD	Range (%)
Rectum	$D_{0.1{\mathrm{cm}}^{3}}$	7.66 ± 1.04	7.1 ± 1.04	7.66 ± 1.17	6.21 ± 0.97	8.3 ± 1.42	8.03 ± 3.31	4.9 to 13.45	0.2 ± 2.21	−1.69 to 3.95	23.77 ± 3.53	20.73 to 29.53	14.6 ± 2.18	11.92 to 18.01	−7.32 ± 3.75	−13.17 to −3.29
	$D_{1.0{\mathrm{cm}}^{3}}$	6.38 ± 0.74	5.76 ± 0.79	6.35 ± 0.85	5.18 ± 0.69	6.75 ± 1.01	11.2 ± 3	9.09 to 16.38	0.72 ± 2.16	−1.2 to 4.39	23.59 ± 4.05	18.85 to 29.07	11.19 ± 3.89	8.05 to 17.83	−5.64 ± 1.59	−7.95 to −3.72
	$D_{2.0{\mathrm{cm}}^{3}}$	5.87 ± 0.73	5.27 ± 0.75	5.84 ± 0.82	4.83 ± 0.65	6.18 ± 0.95	11.87 ± 2.65	9.81 to 16.43	0.81 ± 2.04	−1.01 to 4.26	21.92 ± 3.98	17.56 to 28.07	9.03 ± 4.02	5.26 to 15.78	−5.36 ± 1.41	−7.51 to −3.66

## DISCUSSION

MBDCA is a hot topic in the field of BT. We investigated the dosimetric impact of MBDCA on HDR-BT for cervical cancer using various irradiation techniques. In addition, when the material composition and density of air was assigned to the rectum, the mean difference in the rectum $D_{2{\mathrm{cm}}^{3}}$ between TG-43 and ACE_rec-air_ was 11.92 ± 2.25%, whereas that for the bladder $D_{2{\mathrm{cm}}^{3}}$ was 0.51 ± 1.11%, showing a large difference for the rectum than for the bladder. In addition, the mean difference in the rectum $D_{2{\mathrm{cm}}^{3}}$ between TG-43 and MC_rec-air_ was 21.49 ± 4.47%, whereas that between ACE_rec-air_ and MC_rec-air_ was 8.60 ± 4.64%. The rectum was assigned with air because we wanted to generate a worst-case scenario. Under this condition, ACE_rec-air_ showed smaller differences from the MC simulation (the gold standard) than TG-43. Thus, for patients with considerable air (gas) volume in the rectum, the dose calculation accuracy of ACE may be more accurate than TG-43.

A previous paper has already evaluated ACE for BT, including for patients with cervical cancer. Ma *et al.* evaluated the dose calculation accuracy of ACE for various treatment sites (e.g. prostate and breast) [15]. Standard ACE showed smaller dose differences with MC for the rectum $D_{2{\mathrm{cm}}^{3}}$ than with TG-43 in patients with prostate cancer (−1.33% vs 10.09%). In addition, in patients with breast cancer, the D90 for CTV with ACE was closer to that with MC than with TG-43, which is consistent with our results. Jacob *et al.* reported small differences in DVH parameters between ACE and TG-43 for patients with cervical cancer (<5%) [16]. They assigned the material composition and density of water to the rectum. In ACE_rec-water_, which is the same condition as them, there were small differences in DVH parameters between ACE and TG-43 (<1%). This is consistent with their results. Additionally, we assigned the material composition and density of air to the rectum to generate a worst-case scenario. Our results showed >10% dose difference in the rectum $D_{2{\mathrm{cm}}^{3}}$ between ACE_rec-air_ and TG-43. This difference could be due to different material assignments to the rectum. In addition, the mean difference in the rectum $D_{2{\mathrm{cm}}^{3}}$ between TG-43 and MC_rec-air_ was 21.49 ± 4.47%, whereas that between ACE_rec-air_ and MC_rec-air_ was 4.11 ± 15.78%, suggesting that ACE_rec-air_ provided a more accurate dose distribution than TG-43 when air was used as the rectal material. Thus, for patients with considerable air (gas) volume in the rectum, the dose calculation accuracy of ACE may be more accurate than TG-43. Further investigation is needed to clarify these differences using a larger sample size.

There was no significant difference between the different irradiation techniques, despite using rectum retractors or interstitial needles, possibly because the density of these devices was not excessively high or low compared with that of water (1.0).

## CONCLUSIONS

We investigated the impact of MBDCA (ACE) on HDR-BT for cervical cancer treatment planning. Our results showed that the differences in DVH parameters of TG-43 and ACE were large for the rectum when considerable air (gas) volume was present in the rectum; however, this difference reduced when the air (gas) volume was reduced. In addition, ACE was associated with better dose calculation accuracy than TG-43 in these situations. Thus, ACE may be able to calculate the dose more accurately than TG-43 for HDR-BT when treating patients with cervical cancer, particularly for patients with considerable air (gas) in their rectum.
